# Assessment of treatment response during chemoradiation therapy for pancreatic cancer based on quantitative radiomic analysis of daily CTs: An exploratory study

**DOI:** 10.1371/journal.pone.0178961

**Published:** 2017-06-02

**Authors:** Xiaojian Chen, Kiyoko Oshima, Diane Schott, Hui Wu, William Hall, Yingqiu Song, Yalan Tao, Dingjie Li, Cheng Zheng, Paul Knechtges, Beth Erickson, X. Allen Li

**Affiliations:** 1 Department of Radiation Oncology, Medical College of Wisconsin, Milwaukee, Wisconsin, United States of America; 2 Department of Pathology, Medical College of Wisconsin, Milwaukee, Wisconsin, United States of America; 3 The Affiliated Cancer Hospital of Zhengzhou University, Zhengzhou, China; 4 Cancer Center, Union Hospital, Tongji Medical College, Huazhong University of Science and Technology, Wuhan, China; 5 Sun Yat-sen University Cancer Center, Guangzhou, China; 6 Biostatistics, Joseph. J. Zilber School of Public Health, University of Wisconsin-Milwaukee, Milwaukee, Wisconsin, United States of America; 7 Department of Radiology, Medical College of Wisconsin, Milwaukee, Wisconsin, United States of America; University of Nebraska Medical Center, UNITED STATES

## Abstract

**Purpose:**

In an effort for early assessment of treatment response, we investigate radiation induced changes in quantitative CT features of tumor during the delivery of chemoradiation therapy (CRT) for pancreatic cancer.

**Methods:**

Diagnostic-quality CT data acquired daily during routine CT-guided CRT using a CT-on-rails for 20 pancreatic head cancer patients were analyzed. On each daily CT, the pancreatic head, the spinal cord and the aorta were delineated and the histograms of CT number (CTN) in these contours were extracted. Eight histogram-based radiomic metrics including the mean CTN (MCTN), peak position, volume, standard deviation (SD), skewness, kurtosis, energy and entropy were calculated for each fraction. Paired t-test was used to check the significance of the change of specific metric at specific time. GEE model was used to test the association between changes of metrics over time for different pathology responses.

**Results:**

In general, CTN histogram in the pancreatic head (but not in spinal cord) changed during the CRT delivery. Changes from the 1^st^ to the 26^th^ fraction in MCTN ranged from -15.8 to 3.9 HU with an average of -4.7 HU (p<0.001). Meanwhile the volume decreased, the skewness increased (less skewed), and the kurtosis decreased (less peaked). The changes of MCTN, volume, skewness, and kurtosis became significant after two weeks of treatment. Patient pathological response is associated with the changes of MCTN, SD, and skewness. In cases of good response, patients tend to have large reductions in MCTN and skewness, and large increases in SD and kurtosis.

**Conclusions:**

Significant changes in CT radiomic features, such as the MCTN, skewness, and kurtosis in tumor were observed during the course of CRT for pancreas cancer based on quantitative analysis of daily CTs. These changes may be potentially used for early assessment of treatment response and stratification for therapeutic intensification.

## Introduction

Pancreatic cancer is one of the leading causes of cancer death. In contrast to other gastrointestinal cancers, the outcome remains poor for these patients and has shown no significant improvement during the past 30 years [1]. Currently, chemo-radiation therapy (CRT) is widely accepted in the treatment of locally advanced pancreatic cancer (LAPC) with either resectable, borderline resectable or unresectable disease [2]. Although the current effect of radiation therapy in LAPC has recently been called into question by the international Locally Advanced Pancreatic Cancer (LAP-07) clinical trial [3], this trial also highlights the critical role that advance chemotherapy, radiation therapy delivery modalities and patient selection will play in the future management of LAPC. This makes predicting the treatment responses of the tumor or the organs at risk (OAR) before, or in an early phase during CRT delivery, critical and highly desirable. Such ability to predict outcome would allow personalized adaptive therapy, for example, by modifying the radiation dose and/or spatial dose distribution to account for the patient-specific response, thereby improving overall treatment outcome [4].

CT is by far the most common imaging modality for radiation therapy (RT) planning and image guidance during radiation and for monitoring tumor response to therapy. Traditionally, response assessment has been based upon the simple, and somewhat archaic, geometric variations of the tumor on the CT image. This approach to tumor response assessment is subjective and can underestimate responses in cases with no apparent changes in tumor size. Recent advances in image acquisition and standardization allow quantitative imaging analysis. Radiomics, for example, is an emerging field focused on extracting additional data from medical images through advanced imaging processing and analysis tools [5]. This quantitative data, which extends beyond what is visible to the human eye, has been shown to reveal tumor characteristics and/or treatment responses [6, 7]. For example, it has been reported that CT number (CTN), measured in Hounsfield Unit (HU), can be used to assess radiation effects in lung tumors and normal tissues [8, 9] and in liver cancer [10, 11]. Recently the HU changes in tumor and in select OARs were observed during RT delivery for head and neck cancer based on the daily CTs acquired during the CT-guided RT delivery [12]. As CTs are commonly and frequently used in the daily image-guided radiation therapy (IGRT) for patient positioning [4, 13], the possibility of quantitative use of CT for treatment response assessment represents a convenient approach, which can potentially provide deeper underlying information of the change of disease and assist in response assessment and help to predict outcome during treatment. The quantitative use of CT, e.g., radiomic analysis, has not been investigated for pancreatic cancer. Such a study is desirable considering the critical importance of patient response assessment to local treatment modalities using IGRT. In this study, the HU histogram in the pancreas head (representing gross tumor volume (GTV)) extracted from the daily longitudinal IGRT CT sets collected during IGRT will be quantitatively analyzed. A series of histogram-based radiomic metrics, including mean CTN (MCTN), peak position (PP), volume (total voxels), standard deviation (SD), skewness, kurtosis, energy, and entropy will be investigated to search for the possibility for using these metrics to assess treatment prognosis.

## Materials and methods

The anonymized clinical data collected for 20 patients with tumor in the pancreas head were analyzed retrospectively in this study approved by the Institutional Review Board of Medical College of Wisconsin.

### Daily CT data

The diagnostic-quality daily CTs acquired using an in-room CT (Definition AS Open, Siemens Medical Solutions USA, Malvern) during daily CT-guided RT delivery for the selected 20 patients were analyzed. The patient ages ranged from 56 to 76 with a median of 66. There were 11 males and 9 females. All patients were treated with a radiation dose of 50.4 Gy in 28 fractions with concurrent chemotherapy (mostly Gemcitabine) and 8 of them had 3 to 4 cycles of pre-radiation chemotherapy. The patient characteristics, treatment methods, and treatment outcomes (pathology response, local recurrence and metastatic status) along with other data were summarized in Table 1. All daily CTs were acquired using the same protocol with 120 kVp and a scan resolution of 0.98 mm×0.98 mm in the scanning plane and a 3-mm slice thickness. The GTV (pancreas head), spinal cord and abdominal aorta were contoured using a commercial tool (MIM Software Inc., Cleveland, OH) on the daily CTs. If a stent implanted in the bile duct was seen on the images, the stent was delineated and excluded from the GTV. All contours were delineated manually and reviewed by experienced radiation oncologists. The images and contours were exported from MIM and then analyzed using an in-house program developed in MATLAB (version R2013a; MathWorks, Natick, MA). The CTN of each voxel enclosed by a contour was extracted to generate the histogram. A series of histogram-based radiomics metrics, including the mean HU, peak position, volume, standard deviation, skewness, kurtosis, energy and Shannon entropy were calculated from the HU histogram for each contoured structure on each daily CT. The CTN time stability of the CT scanner was checked in a monthly QA procedure with a standard CT phantom for a period of 40 months, covering the treatment time for all patients analyzed in this study. The standard deviation of the MCTN of water is 0.49 HU with a range of the change from -1.0 to 1.1 HU throughout the entire period of inspection, indicating the small noise level and high stability of the scanner.

**Table 1:** Patient characteristics, treatment methods and outcome data.

Patient	Gender	Age (y)	Tumor Stage	Pre-radiation Chemo	Concurrent Chemo	Pathology Response Grade	Local Recurrence/ Metastatic Status	ΔMCTN (HU)
1	F	59	resectable	No	Gemcitabine	NA	No/Yes	-5.5
2	F	58	resectable	No	Gemcitabine	3	No/Yes	-1.1
3	M	56	bordline resectable	Yes	Gemcitabine	NA	No/No	-3.6
4	F	65	resectable	No	Gemcitabine	1	No/Yes	-5.8
5	F	76	resectable	No	Gemcitabine	NA	No/No	-8.2
6	M	75	bordline resectable	Yes	5-FU	1	No/No	-15.8
7	M	58	nonresectable	No	Capecitabine	NA	No/No	0.1
8	M	59	resectable	No	Gemcitabine	NA	No/Yes	-3.2
9	M	76	resectable	No	Gemcitabine	0	No/No	-8.8
10	F	76	nonresectable	Yes	Capecitabine	0	Yes/No	1.0
11	M	72	resectable	No	Gemcitabine	1	No/No	-8.0
12	F	74	resectable	No	Gemcitabine	1	No/No	-8.1
13	F	57	bordline resectable	Yes	Capecitabine	2	No/Yes	-4.1
14	M	75	bordline resectable	No	Gemcitabine	1	No/No	-0.3
15	M	58	bordline resectable	Yes	Gemcitabine	3	Yes/Yes	-9.2
16	M	56	bordline resectable	Yes	5-FU	2	No/No	3.9
17	F	69	bordline resectable	Yes	Gemcitabine	2	No/No	-0.7
18	M	65	resectable	No	Gemcitabine	3	No/Yes	-4.8
19	F	66	resectable	No	Capecitabine	3	Yes/No	-4.9
20	M	64	bordline resectable	Yes	Gemcitabine	1	No/No	-7.2

NA: Not applicable as no surgery was performed for the patient.

### Pathologic data

Out of the 20 patients, 15 underwent pancreatectomy after CRT with gross and microscopic pathology reported. The surgical specimen was fixed in formalin overnight and then the pancreas was serially sectioned. The area of tumor and surrounding fibrosis in the pancreas was submitted for microscopic examination. Hematoxylin and eosin sections were prepared and treatment effect was evaluated. A modified Ryan Scheme for tumor regression score recommended by the College of American Pathologists was used to evaluate treatment effect as follows: Grade 0: no viable cancer cells (complete response), Grade 1: single cells or small groups of residual cancer (near complete response), Grade 2: residual cancer with evident tumor regression, but more than single cells or rare small groups of cancer cells (partial response), and Grade 3: extensive residual cancer with no evident of tumor regression (poor or no response) [14].

### Data analysis

The change in each CT metric between fractions was evaluated with the student’s paired two-tailed *t*-test. The difference of trends between the pathological response groups were tested with the GEE model [15]. Spearman and Pearson correlation were used to quantify the association between CT metrics. For all statistical analyses, p < 0.05 was considered significant.

## Results

### Average changes of radiomic metrics during chemoradiation therapy

**Fig 1:**
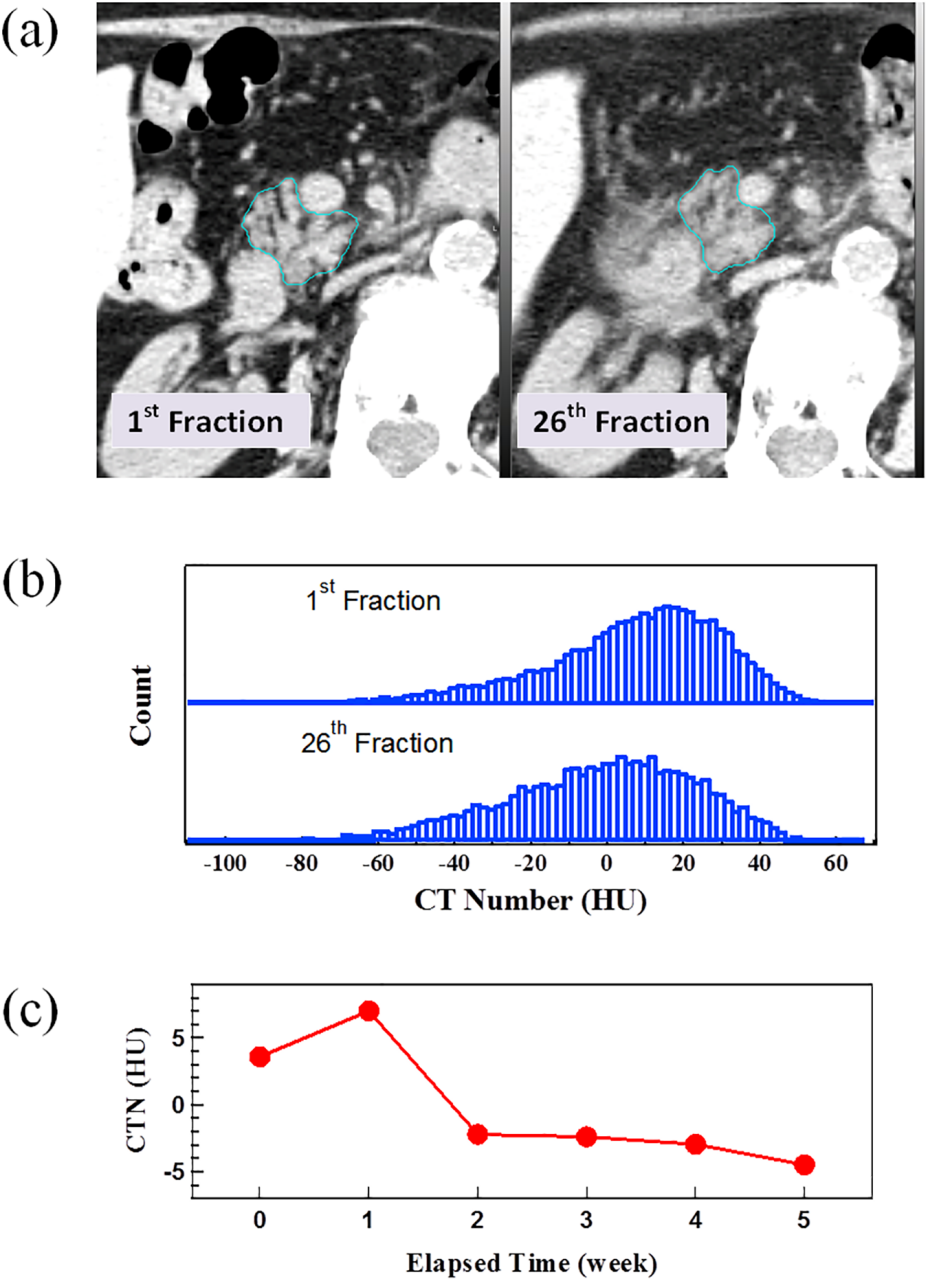
An example case. (a) a comparison of axial CT slice with contour of the GTV (pancreas head) from the daily CTs for the 1^st^ and 26^th^ fractions, (b) HU histograms of the GTVs for the 1^st^ and 26^th^ fraction (the histograms were normalized and the actual ratio of volumes was 1.73:1), and (c) the change of mean CTN in GTV with elapsed time through the course of treatment.

Fig 1 presents the analysis for a representative patient, including (a) a comparison of daily CTs between the 1^st^ fraction when the patient did not receive any irradiation and the 26^th^ fraction following irradiation of 25 fraction x 1.8 Gy in 5 weeks, (b) a comparison of CTN histograms of GTVs from the 1^st^ and 26^th^ fraction CTs and (c) the change of GTV MCTN during RT delivery (elapsed time). The GTV contours in Fig 1(A) enclose the entire pancreatic head excluding the stent and blood vessels. The CTN histogram in Fig 1(B) shows a single asymmetric peak skewed to the high HU direction, with the peak shifting to low HU direction as a result of irradiation. In addition, the peak becomes broadened and flat after the treatment. Consequently, the GTV MCTN decreases with the increase of elapsed time (Fig 1(C)).

**Fig 2:**
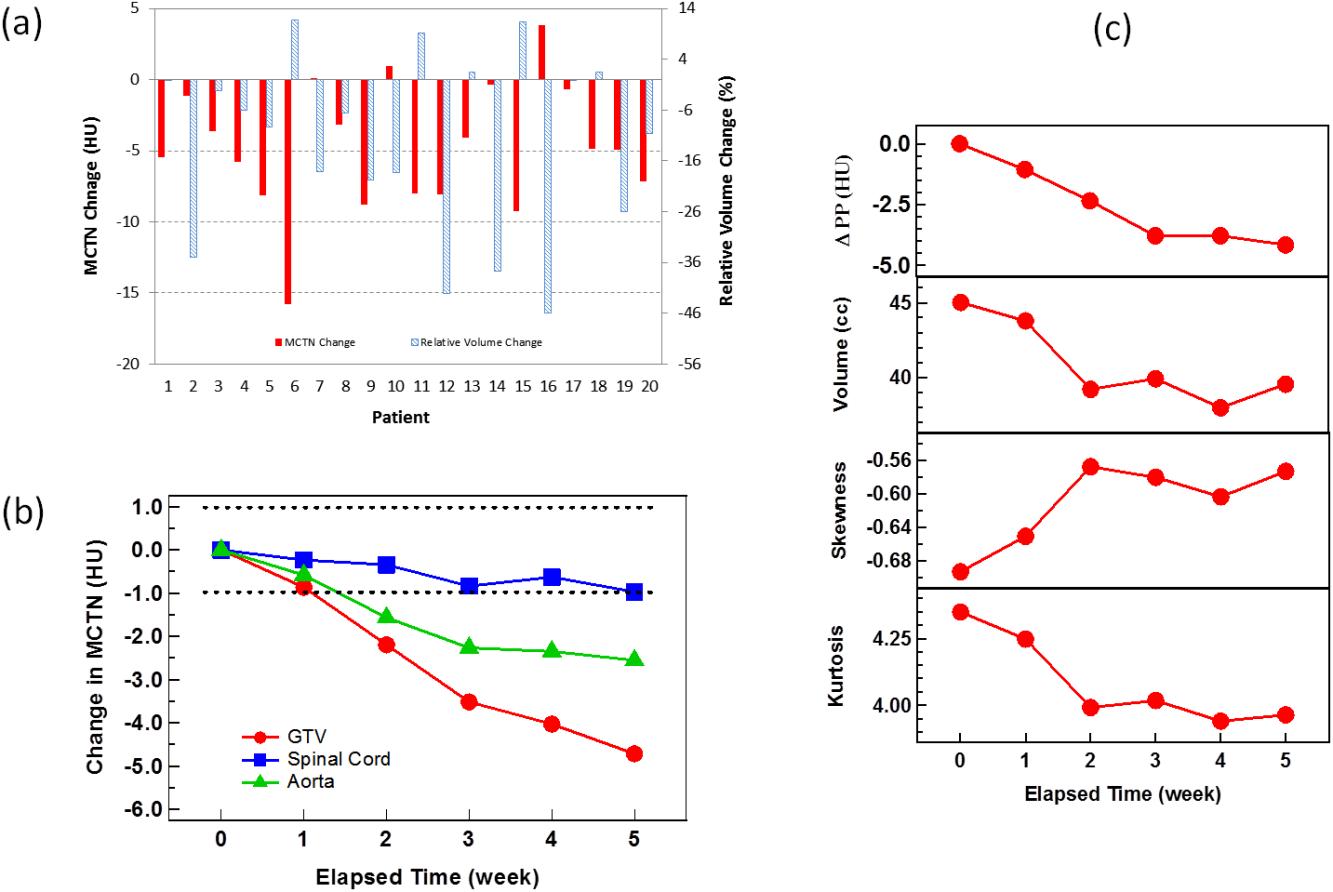
Changes in MCTN and other metrics with treatment. (a) The MCTN (solid red) and relative volume (dashed blue) changes from the 1^st^ fraction to the 26^th^ fraction for the 20 patients; (b) the average MCTN changes over all patients with respect to the elapsed time of treatment for the GTV, spinal cord, and blood in aorta. The uncertainty region (shaded) of the machine measurement was defined with the 95% confidence interval within the ±2σ dash lines determined from phantom measurement over 40 months; (c) the average changes in PP, volume, skewness, and kurtosis over all patients with respect to the elapsed time of treatment for the GTV.

Similar trends described above were generally observed for all patients. The GTV MCTN at the beginning of the treatment (1^st^ daily CT) ranged from 3.4 to 33.7 HU with an average of 17.9±9.2 HU. During the course of treatment delivery, the MCTN generally decreased with the increase in elapsed time. For all of the patients, the changes in GTV MCTN and relative GTV volume from the 1^st^ to 26^th^ fraction are shown in Fig 2(A). Average MCTN reduction from the first to the 26^th^ fraction is 4.7±4.5 HU, ranging from -15.8 to 3.9 HU. The relative changes of MCTNs in GTV (pancreas head), spinal cord and aorta during the course of CRT averaged over all the patients are shown in Fig 2(B). After one week of treatment, the average change of MCTN for the GTV fell outside the uncertainty region (shaded region), which quantifies the MCTN variation due to the machine instability as determined from the monthly QA. As the radiation dose to the spinal cord is generally much lower than that to the GTV and within the machine repeatability noise range, it is reasonable to use the change in the MCTN of the spinal cord as a control. The pancreatic body or tail was not selected as a control as both were generally irradiated with the similar radiation dose level as for GTV. Although the average MCTN change observed in GTV was not drastically large compared to the noise, it is however significant (further statistical proof will be provided in the next paragraph) and was introduced by radiation. Furthermore, as shown in Fig 2(A), the changes are patient specific and 9 out 20 patients (45% of the total cohort) had a MCTN decrease greater than 5 with a maximum of 15.8 HU. Similar to MCTN, other HU histogram metrics, such as PP, volume, skewness, and kurtosis demonstrate corresponding changes during RT delivery, as shown in Fig 2(C) for the average values over all 20 patients.

The p-values from the student’s paired two-tailed *t*-tests for the changes of MCTN, PP, volume, SD, skewness and kurtosis for GTV and MCTN for the spinal cord and aorta between the 1^st^ fraction and the 2^nd^, 3^rd^, 4^th^ and 5^th^ weeks are shown in Table 2. Major changes in various metrics during the RT delivery, as observed from Fig 2 and Table 2, are described below. The changes in MCTN, PP, volume and kurtosis for GTV become significant following the second week and throughout the remaining weeks; the peak position of HU histograms shifted towards lower HUs; the GTV (pancreas head) volume reduced, from the average volume of 45.0±29.0 cc before the treatment with an average reduction rate of 4.2%±12.6% in a week and 12.2%±17.7% in 5 weeks. After 5 weeks, there were 15 out of 20 cases showing volume decreases with the maximum reduction of 45.9% and 5 cases showing increases with the maximum enlargement of 11.6%. The sharpest drop occurred at the beginning of the third week. The skewness increased from -0.69 to -0.57, indicating that the HU histogram was left-tailed originally and became less skewed after the treatment. The kurtosis decreased from 4.35 to 3.96. Compared to a normal distribution, which has a kurtosis of 3.0, the distribution was heavy-tailed and became less tailed at the end of treatment. Similar to the volume drop, the sharpest drop of kurtosis also occurred at the beginning of the third week. The average SD, energy, and entropy for GTV were also calculated but these metrics did not change significantly on average during the course of CRT. A very strong correlation was found between the values of SD and energy (Spearman test, ρ = -0.99, p<0.00001), and SD and entropy (ρ = 0.99, p<0.00001), indicating that these metrics are mathematically related to each other.

**Table 2:** Results of student’s paired two-tailed *t*-tests between the first week and the remaining weeks of the treatment for 6 histogram metrics as well as MCTNs for the spinal cord and the blood in the abdominal aorta. The significant results are bolded.

	2^nd^	3^rd^	4^th^	5^th^	6^th^
MCTN (GTV)	0.18	**0.025**	**0.0004**	**<0.0001**	**<0.0001**
MCTN (Cord)	0.57	0.51	0.075	0.27	0.095
MCTN (Blood)	0.28	**0.011**	**0.00064**	**0.0019**	**0.0013**
PP	0.19	**0.015**	**0.0004**	**0.0014**	**0.0022**
Volume	0.12	**0.006**	**0.005**	**0.001**	**0.012**
SD	0.67	0.21	0.63	0.73	0.63
Skewness	0.26	**0.003**	**0.029**	0.095	**0.011**
Kurtosis	0.23	**0.011**	**0.029**	**0.027**	**0.009**

### Patient specific changes of radiomic features during CRT

**Fig 3:**
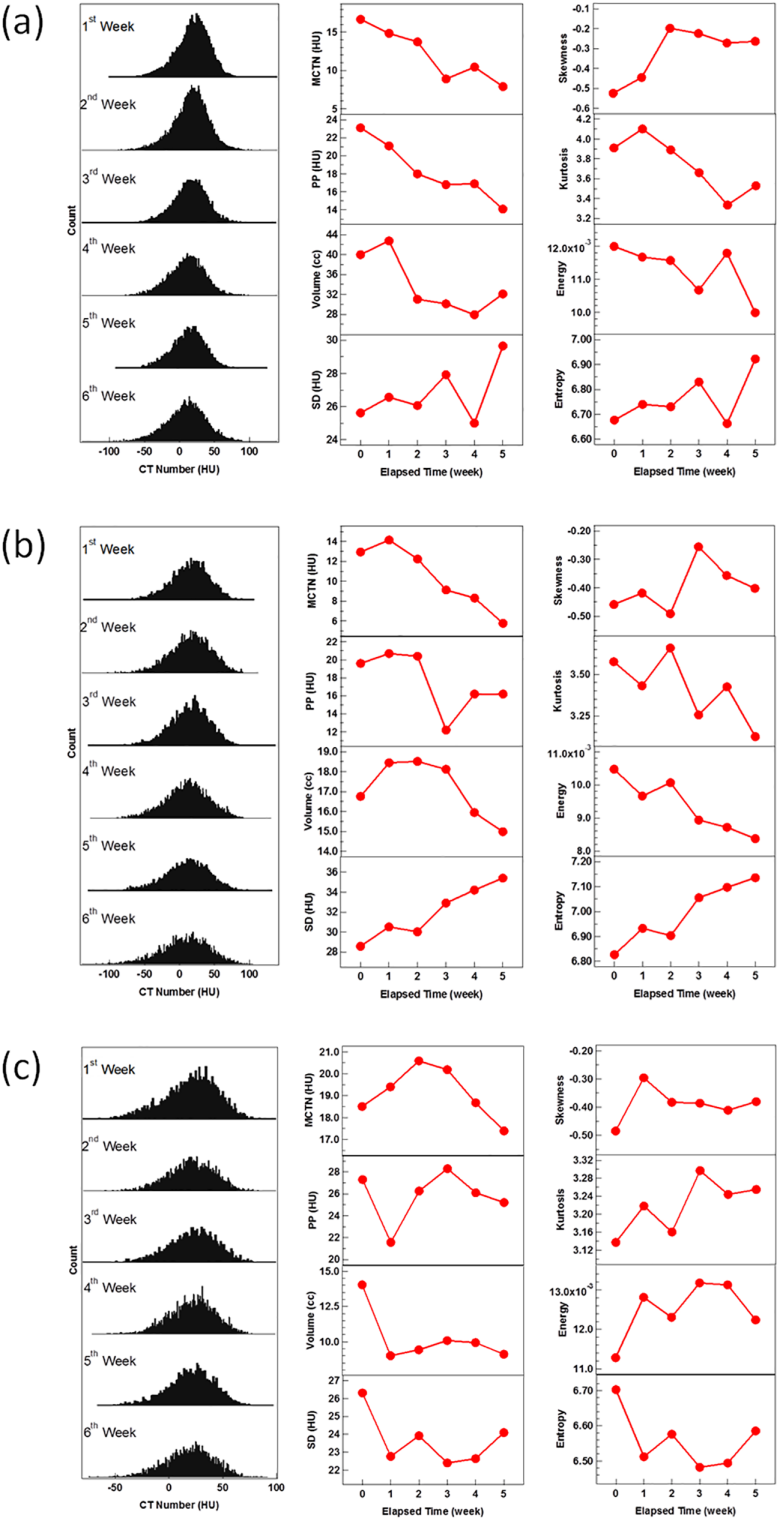
The changes in CTN histograms and metrics for three typical patients. The CTN histograms of the GTVs for the first day of each week along with the changes of the eight metrics with respect to the elapsed treatment time for two good pathologic response patients: (a) Patient 9 and (b) Patient 20, and for one with poor response (c) Patient 3.

The detailed changes in radiomic metrics during the course of CRT were observed to be quite patient specific. To demonstrate, Fig 3 presents changes of various radiomic metrics for three cases, with two cases (a and b) having a good and one (c) having a poor pathologic tumor response after CRT. For the two cases with a good response, the MCTN dropped by 8.9 and 7.2 HU within 5 weeks, respectively. The SD, entropy and skewness exhibited increasing trends. For the case with poor response, the MCTN was reduced by 1.1 HU during the treatment. The changes in SD, entropy, and skewness were small and lacked a clear trend.

### Association between radiomics change and pathologic response

**Fig 4:**
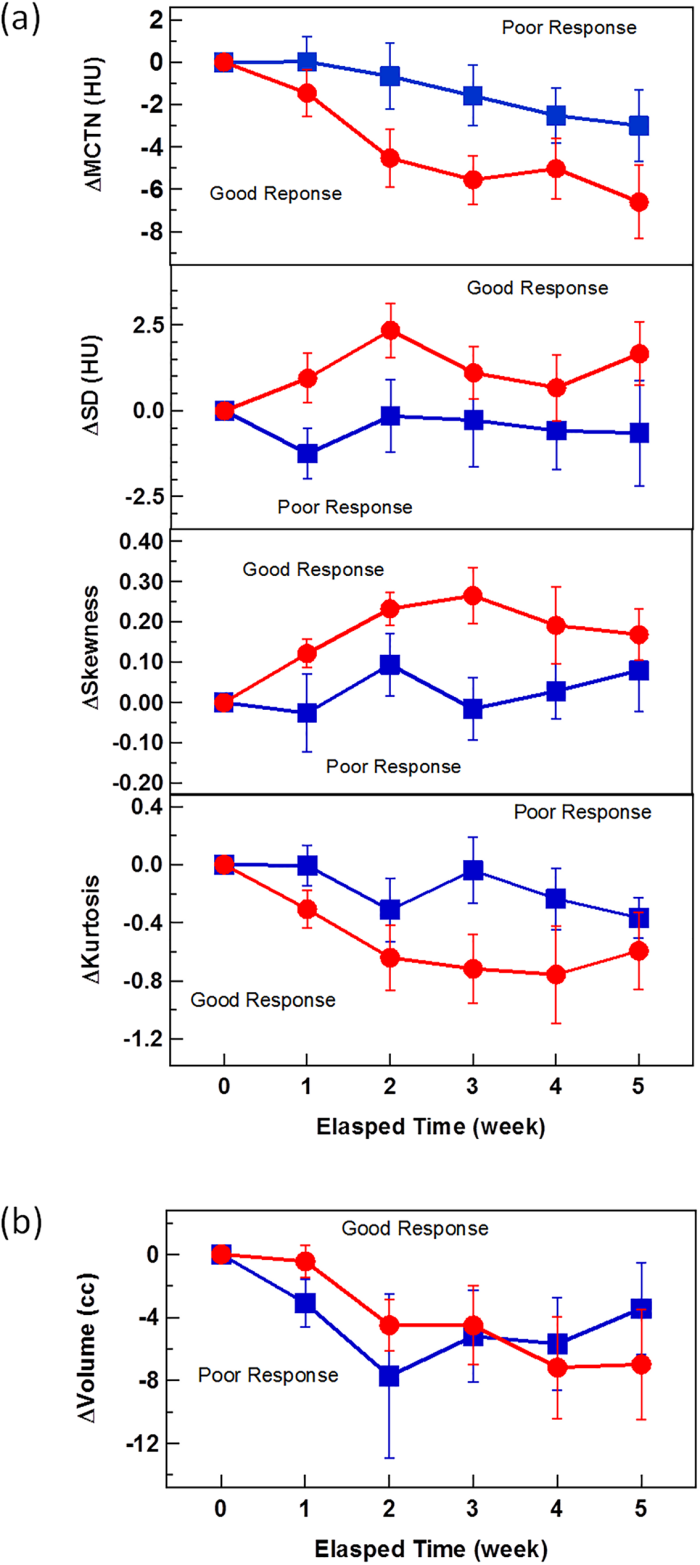
Comparisons of the average changes of for the good- and poor-response groups. (a) moments of histogram including MCTN, SD, skewness, kurtosis, and (b) volume in GTV from the first RT fraction during the course of CRT. The error bar is the standard error of the values of the cohort.

Among the 20 patients studied, 15 underwent surgery after CRT. Nine out of these 15 patients had a good tumor response (Grade 0 or 1) and the other 6 had a poor response (Grade 2 or 3). Fig 4 compares the average changes of (a) MCTN, (b) SD, (c) skewness, and (d) kurtosis in GTV from the first RT fraction during the course of CRT for the good- and poor-response groups. The average changes of MCTN, SD, skewness, and kurtosis from the first to the last RT fractions are -6.6/-3.0 HU, 1.7/-0.7 HU, 0.17/0.08, and -0.37/-0.59, respectively, for the good and poor response groups. As indicated in Fig 4, the changes in the HU histogram features are different and separated with treatment time between the good and poor pathological response groups. The patients with a good response tend to have large decreases in MCTN and skewness and larger increases in SD and kurtosis than those with a poor response. The p values for the separation, as obtained from the GEE model fit, are 0.046, 0.058, 0.042, and 0.12 for MCTN, SD, skewness and kurtosis, respectively. In contrast, similar trends were not observed in the volume changes. These different changes can be observed as early as in the first few weeks during the course of CRT.

### Association between initial value and change of MCTN

**Fig 5:**
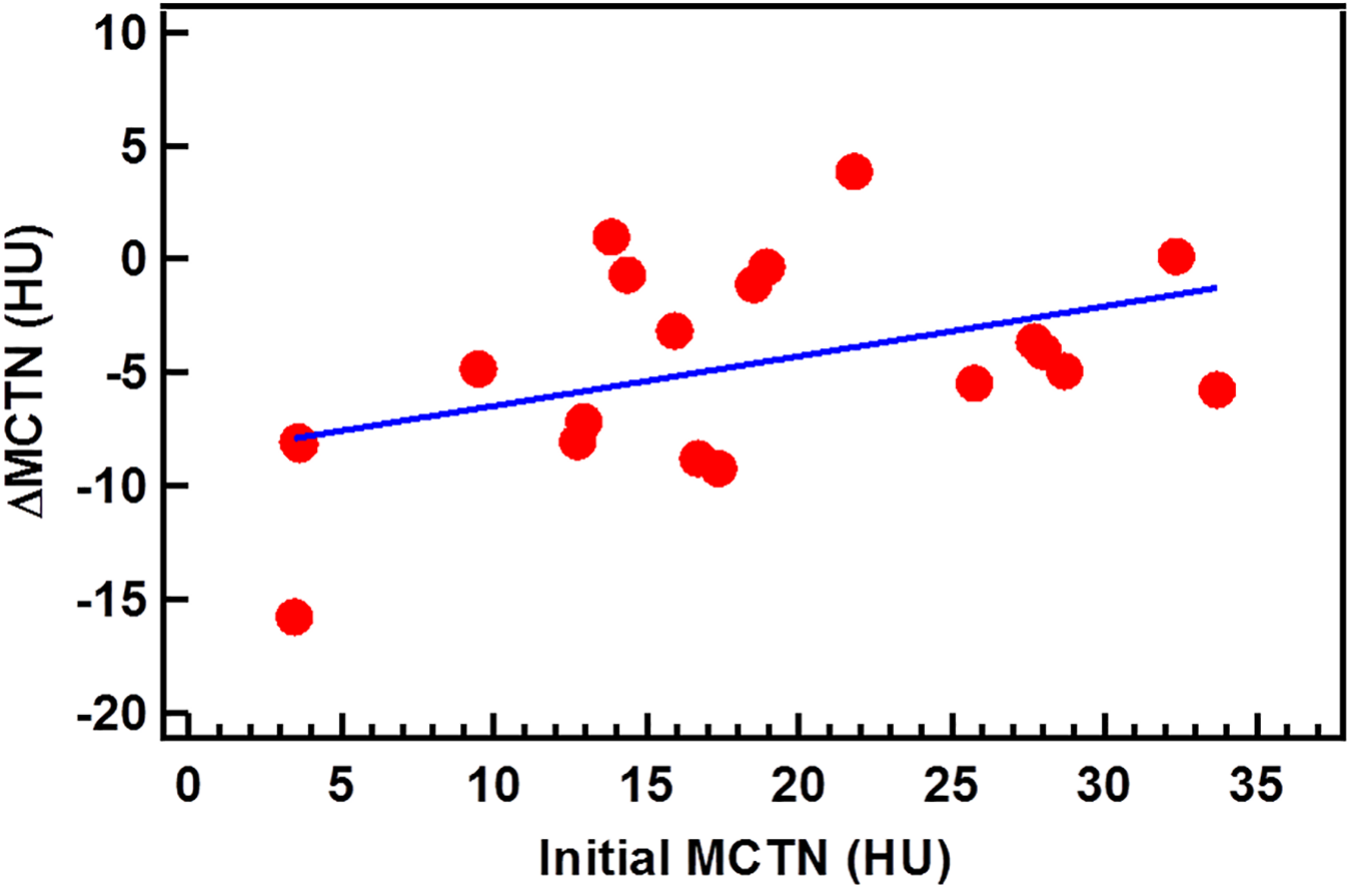
The correlation of the change and initial value of MCTN. The straight line is the best linear fit.

The MCNT change from the 1^st^ to 26^th^ fraction is presented as a function of the initial MCTN in Fig 5 for the 20 patients. The correlation examined with a Pearson test shows a coefficient of 0.45 (p = 0.046), suggesting a moderate correlation between these two.

## Discussion

Radiation induced CTN changes in tumor and/or normal tissue have been reported previously for several tumor sites, including lung [8, 9], liver [10], brain [16], testicle [17], and head and neck [12], although such changes were not seen for prostate and bladder cancers [18, 19]. The changes vary with sites, radiation dose, and different types of tissues. Lung cancer irradiation led to a mean decrease of 18.9 HU in MCTN of tumor [9]. A radiation-induced MCTN decrease of larger than 13 HU was associated with local control while the tumor volume change was not [9]. MCTN change was also used to analyze the radiation-induced damage of normal lung tissue, particularly for stereotactic body RT (SBRT) [8, 20]. For normal liver tissue, the irradiated region was reported to have a MCTN change of 15 HU less than the non-irradiated region for 61 out of 95 liver cancer patients undergoing SBRT [11]. The average MCTN change of patients demonstrated a linear decrease with a slope of -0.65 HU/5 Gy [10]. A decrease of 5 HU in MCTN was observed in the white matter after brain irradiation [16]. For head and neck tumors, the MCTN of the tumor was reduced by 5.9 HU after treatment of 70 Gy in 35 fractions while the MCTN of the ipsilateral parotid gland tended to have a larger decrease (11.0 HU) than that of the contralateral parotid gland (6.8 HU), manifesting radiation dose effect [12].

In this study, the MCTN in GTV (pancreas head) drops by as much as 15.8 HU during CRT with an average reduction of 4.7 HU for the patients studied. One of the reasons for the small average change may be partially due to the lower radiation dose for pre-operative irradiation of pancreas cancer as compared to other tumor sites. The average MCTN change, although small, is significant and comparable to the change reported for head and neck cancers [12] and for the white matter in the brain [16]. Furthermore, the MCTN reduction greater than 5 HU was seen in 45% of cases and correlates with better pathological response. These imply that the low MCTN reductions observed for some cases are due to their poor pathological responses to the CRT, not due to the measurement uncertainties.

The MCTN and volume changes may measure different aspects of treatment response and can be obtained from the histogram analysis. In this exploratory study, MCTN was found to be more indicative than volume as demonstrated in Fig 4.

The underlying mechanism of the radiation-induced changes in CT radiomic metrics is not fully understood. Roughly speaking, RT can result in tumor death which leads to changes in tissue perfusion. When the tumor cells are dead and replaced by fluid or normal cells, the tumor density goes down and so does the MCTN. Mathematically, the observed MCTN reduction is due to the shift of the HU histogram. The correlation between the change in MCTN and shift of PP was checked with a Pearson test. A coefficient of 0.63 suggests a fair correlation of these two parameters (p = 0.003). The fact that the correlation is not very strong indicates the shift is only one of the factors affecting the MCTN change. In fact, the shape of the peak represented by SD, skewness and kurtosis, is also altered, which suggests the corresponding components change as well. Because tumor and healthy tissue in the pancreatic head have similar electron densities and can be difficult to distinguish based on CT, they are both enclosed in the GTV contour which is the entire pancreatic head. Either of them may continue to be divided into more parts. The non-Gaussian histogram distribution at the beginning of the treatment is evidence of tissue inhomogeneity. It is very likely that different components react differently to radiation and some of them respond more quickly and more dramatically than others. All these differences inevitably contribute to the shape change of the histogram, usually a spread of the peak as indicated by the changes in SD, energy, and entropy. On the other hand, changes in both skewness and kurtosis suggest that the CTN histogram gradually transforms to a Gaussian distribution. This implies that some parts, most likely tumor, shrink or even disappear as the treatment proceeds.

This hypothesis was suggested by other studies [9, 12]. For lung tumors, two components, tumor and edema/necrosis, were assigned to the GTV and the MCTN change is attributed to the transition from tumor to edema/necrosis [9], which was supported by an animal study [21]. The multiple components in the GTV was also evident in the CTN change in head and neck tumors where two types of CTN histograms were observed [12], where one had a narrow peak and the other had a broad skewed peak. The former disappeared completely with radiation, the latter split into two peaks, and one of them remained till the last fraction without substantial change.

Among many other factors that can contribute to the radiation-induced CTN change, the ubiquitous presence of blood may be one [22]. It has been suggested that an increase in blood volume might result in CTN change [12]. Increases of blood volume for locally controlled tumors were significantly higher than for those with local failure [23]. A change in CTN can thus be the result of an increase in the tumor blood volume and can be correlated to the prognosis. Besides the volume effect of blood, the CTN of blood itself can also change during treatment and should be considered. As shown in Fig 2(B), the MCTN of blood in the aorta decreases as the treatment proceeds. This decrease may be due to the chemoradiation-induced anemia. It is well understood that the radiodensity correlates with the hemoconcentration [24]. CRT can cause damage to red bone marrow and result in decrease in the number of blood cells, which causes the CTN reduction in blood [25]. This radiation-induced blood effect can lead to the decrease of CTN in the GTV as well as other tissues involved in the blood supplies. The GTV CTN drop caused by the blood CTN drop can vary depending on the change of blood volume in GTV. The blood CTN change, however, was not as high as the GTV CTN, indicating there are other mechanisms for the CTN change, such as the direct response of tumor cells.

Although currently the exact mechanism is not clear, changes in CTN as well as other radiomic metrics may precede radiographic tumor changes and be used as indicators for tumor response. Specifically, patients with a good response tend to have large reductions in MCTN and skewness, and large increases in SD and kurtosis. These metrics could be used to help identify patients who are responding or not responding to the treatment. For a non-responding tumor, the treatment may be adapted to address the lack of response. The correlation between the MCTN change and the initial MCTN demonstrated in Fig 5 implies that the physical and biological properties of patient anatomy can play a role in the treatment response prediction. If this finding is confirmed with large patient data sets, the initial MCTN along with other radiomic features may be used for screening and/or treatment selection.

It should be noted that there are many other factors that can affect the analysis due to the complexity of the disease and treatment. One of these factors is the time gap between the RT and the surgery. For the cohort in this study, the average RT-surgery span is 34 ± 6 days. Although the dispersion is narrow, there is a moderate correlation between the tumor response and the length of RT-surgery span (Spearman test, ρ = 0.58, p = 0.03). The difference can alter the pathology results and can increase the complexity of the correlation analysis.

Another influencing factor is chemotherapy. The overall tumor response results from radiation as well as chemotherapy. These two effects cannot be separated as there is no data for radiation alone available thus they were dealt as a whole in this study. Before RT, 8 of the patients were treated with pre-radiation chemotherapy and 12 patients without. Between the two groups, no significant difference was found in the initial and final MCTN (student’s two-tailed *t*-test, p = 0.8, 0.8, 0.9). No association was found between the MCTN changes and the local recurrence and metastatic status. This may be because (1) these clinical outcome can be influenced by many factors, including the effects of pre- and post-CRT treatments; and (2) the data size included is too small and the patient population is too heterogeneous.

It should also be noted in most of the cases studied, we have observed on pre-CRT CTs (e.g., CTs at the first fraction) that the surrounding normal pancreatic tissues generally have slightly lower HUs than the tumor in pancreas head. There are also measurable differences in some radiomic features (e.g., entropy). However, these differences are generally too small to distinguish the tumor region from the surrounding normal pancreatic tissue. In a separate study, we are investigating these differences with help from MRI.

There are two major limitations of this exploratory study. One is the small patient cohort. There are only 15 out of the 20 patients who had pathology data. This small number prevents us from obtaining a solid correlation between radiomic change and treatment response. The other limitation is the defined GTV encloses both tumor and normal pancreas tissue because they are not distinguishable on CT. This would lead to difficulty and inaccuracy in tracking real changes in tumor volume and shape during the course of treatment. Since their responses to irradiation are most likely different, the measurement of tumor treatment response may be dampened. In addition, the contouring variation, such as that due to the subjective judgment on where the pancreatic head begins and ends, may also affect the analysis, even though, the contouring variation was minimized by carefully reviewing the consistency of daily contours with an experienced radiation oncologist.

## Conclusions

The daily diagnostic-quality CTs acquired during CRT for pancreatic cancer were quantitatively analyzed by extracting a series of intensity histogram based radiomic metrics. Significant changes in various metrics, including MCTN, PP, volume, SD, entropy, skewness, and kurtosis were observed for most of patients studied. These changes are patient specific and can be observed as early as in the first few weeks during the course of treatment. Patients with good pathologic tumor response tended to have large changes in GTV MCTN, SD and skewness as compared to those with poor tumor response. With further studies, these radiation-induced changes in radiomic features may potentially be used for early assessment of treatment response during radiation delivery.
